# Changing the course of comorbid eating disorders and depression: what is the role of public health interventions in targeting shared risk factors?

**DOI:** 10.1186/2050-2974-2-15

**Published:** 2014-05-27

**Authors:** Carolyn Black Becker, Maribel Plasencia, Lisa Smith Kilpela, Morgan Briggs, Tiffany Stewart

**Affiliations:** 1 Department of Psychology, Trinity University, San Antonio, TX 78212, USA; 2 Pennington Biomedical Research Center, 6400 Perkins Rd, Baton Rouge, LA 70808, USA

**Keywords:** Eating disorders, Depression, Prevention, Public health interventions, Risk factors, Obesity

## Abstract

Public health has a productive history of improving global health due to its focus on reaching large populations using effective and scalable interventions. Yet, the marriage between evidence-based science and the implementation of community/public health interventions within mental illness remains underdeveloped. Research suggests that major depression is the most commonly cited comorbidity for eating disorders (EDs). Thus, identification of public health strategies that jointly impact depression and EDs, including shared risk factors, has the potential to significantly impact mental health suffering. The primary aim of this paper is to examine and discuss such public health approaches as well as explore cues taken from public health efforts to inform future directions in research and clinical practice. As a comprehensive review of all public health initiatives that address EDs and depression is beyond the scope of this paper, this paper reviews a series of programs/approaches that either are of large scale and/or have received empirical support. In particular, public health related interventions that aim to reduce variable risk factors associated with EDs and depression, as well as interventions that aim to reduce continuous measures of ED and depression symptoms are reviewed. To date, despite significant progress in modifying risk factors for EDs and depression, the field still lacks a public health study that has been appropriately designed and/or adequately powered to assess true ED/depression prevention effects. Further, although several programs show promise, many widely disseminated approaches lack empirical support, raising concerns about the potential for waste of limited resources. In summary, although the combination of prevention and public health based approaches appear to have merit when trying to move the needle on risk factors and symptoms associated with EDs and/or depression, further research is needed to investigate the reach and effectiveness of large scale dissemination efforts of such endeavors.

## Introduction

Despite significant advances within the mental health field in the development and testing of treatments for mental illness, the distance between scientific findings and community-based implementation of evidence-based interventions remains sizeable [1-3]. Moreover, Kazdin and colleagues have convincingly argued that the dominant delivery mode of intervening with mental illness (i.e., face-to-face psychotherapy provided by a highly trained masters or doctoral level therapist) will never be sufficient to meet the needs of those suffering from mental illness due to a variety of factors (e.g., insufficient number of trained therapists, cost, geographical challenges) [1,4]. As such, they called on the mental health field broadly to increase the range of models for delivering mental health services. One proposed strategy involves increased collaboration with other disciplines in moving beyond a focus on standard one-on-one psychotherapy [1]. The aim of this paper is to investigate to what degree current research supports the adoption of strategies drawn from public health with the aim of impacting the course of comorbid eating disorders (EDs) and depression through the targeting of shared risk factors and to make suggestions for future directions, both clinically and empirically. We focus on EDs and depression because public health initiatives that can address these dual issues are likely to have greater effect in reducing mental health suffering.

Public health appears to offer a tremendous resource to the ED and depression fields in our efforts to reduce mental illness suffering [5]. More specifically, public health interventions have a longstanding history of contributing to health on a national and international scale. For example, throughout the 20th century, the field of public health improved the United States’ population health, adding 25 years to the nation’s average life expectancy [6]. Important public health achievements, which were not limited to the United States, during this time included: immunizations, motor-vehicle safety, control of infectious diseases, food safety, prenatal care, fluoridation of drinking water, and identification of tobacco use as a hazardous health behavior [6]. Of note, these public health initiatives evidenced high scalability (i.e., interventions could be “scaled up” and delivered to large populations without losing effectiveness); thus the impact of such initiatives was far-reaching and change observable across the population.

Furthermore, as illustrated by the aforementioned examples, the field of public health provides a myriad of strategies and perspectives for widespread impact regarding health behaviors and illness prevention. Although a variety of definitions exist, most public health approaches to behavior change include four main concepts: 1) a focus on population health and public policy; 2) emphasizing promotion and prevention; 3) addressing determinants of health (i.e., risk factors); and 4) engaging in a three-step action plan (i.e., gathering data, creating policies, and ensuring policies are implemented and enforced) [7]. These concepts are evident in an array of existing public health initiatives targeting mental illness.

Written from the perspective of the eating disorder field, the aim of this paper is to address public health efforts that could impact comorbid EDs and depression. We first briefly review the literature on comorbidity. We then discuss shared variable risk factors (i.e., risk factors that have the potential to be changeable [8]) for EDs and depression given that, to our knowledge, no public health program/intervention exists that targets both clinical ED and depression diagnoses. As such, we focus on a variety of public health related interventions that aim to reduce variable risk factors associated with EDs and depression, as well as interventions that seek to reduce continuous measures of ED and depression symptomatology.

## Review

### Eating disorders and depression: comorbidity

The term comorbidity describes the situation in which a given individual meets criteria for more than one disorder. Thus, in the case of EDs and depression, comorbidity rates describe the percentage of a given population that meets criteria for both disorders. Research suggests that EDs and depression are highly comorbid [9-11]. For instance, Green and colleagues found that lifetime prevalence rates of major depression in patients diagnosed with EDs ranged from 36% to 86% [9]. It is important to note, however, that other disorders also are highly comorbid with EDs. For instance, although studies generally reveal higher rates of depression as compared with anxiety disorders in anorexia nervosa [12-15], researchers have used the high rates of anxiety disorders in EDs to argue against a specific relationship between EDs and depression [12]. Further, Schwalberg and colleagues [16] found no significant difference between the lifetime prevalence rates of depression between individuals with bulimia nervosa, social phobia, or panic disorder, suggesting that the connection between depression and EDs may be nonspecific.

It also can be helpful to examine the association of continuous measures of depressive and ED symptoms (versus rates of comorbid full syndrome disorders), particularly in younger populations given that they are often a primary target for public health prevention approaches. Research here again supports a link. For instance, in a sample of 241 male and female high school students Santos, Richards, and Bleckley [17] found disordered eating attitudes and depressive symptoms to be positively correlated such that as disordered eating attitudes increased, depressive symptoms also increased. Moreover, results indicated that disordered eating attitudes accounted for significant variance in self-reported depressive symptoms even when statistically controlling for the effects of body dissatisfaction, social support and self-esteem [17]. In summary, significant research supports the comorbid relationship between EDs and depression at both clinical and sub-threshold levels.

### Shared variable risk factors

As noted above, one of the common components of public health approaches is a focus on determinants of health, or risk factors for illness. The risk factor approach identifies an antecedent condition and its association to a subsequent outcome. This approach aims to identify factors that increase the probability of a negative outcome compared to the rate of a control population. Additionally, researchers may identify protective factors that increase the probability of a positive outcome or decrease the probability of a negative outcome. Although it is beyond the scope of this paper to conduct a comprehensive review of shared risk factors for depression and EDs, we provide an overview of some major commonalities in this area. Notably, we focus on potentially modifiable risk factors, or what have been referred to as variable risk factors versus fixed markers [8]. Fixed markers are risk factors that cannot be changed. For instance, being female is a very potent risk factor for both EDs and depression. Yet, because one cannot truly alter gender (i.e., we can only alter appearance of gender with surgery and hormones), it is not a useful risk factor when it comes to intervening with EDs or depression except with respect to identifying higher risk populations.

Described by Jacobi and Fittig [18] as one of the most potent and best confirmed ED risk factors, body dissatisfaction (including weight concerns/shape concerns) also increases risk for depression. Indeed, research indicates that body dissatisfaction prospectively predicts a host of negative outcomes including increases in low self-esteem, depression, binge eating, emotional eating, use of unhealthy weight control behaviors, decreased physical activity, and, among overweight girls, increased weight gain between 1–5 years later [19-21]. One model that accounts for the association between body dissatisfaction, EDs and depression has been mapped out by Stice, Nemeroff, and Shaw [22]. The dual pathway model posits that internalization of the thin-ideal standard of female beauty increases risk for body dissatisfaction which in turn increases risk for both dietary restraint and negative affect (i.e., depressed mood), both of which in turn increase risk for ED symptomatology. Research also suggests that the relationship between dieting and depressed mood may interact to increase risk for EDs. For instance, Isomaa, Isomaa, Marttunen, Kaltiala-Heino, and Björkqvist [23] found that dieters who were also depressed and dieters who endorsed “feeling fat” had a 15-fold increased risk of developing a lifetime ED as compared to “vanity” (i.e., dieting to achieve the thin-ideal) or “overweight” (i.e., dieting to reduce negative health outcomes associated with being overweight) dieters.

As described below, the dual pathway model and associated research linking cultural pressures to achieve the thin-ideal standard of beauty, thin-ideal internalization, body dissatisfaction, negative affect, dieting, and ED symptoms have driven many public health-based attempts to target the course of EDs and depressed mood. It is important to note that additional research by Stice and colleagues supports a more complex multidirectional relationship between these variables, rather than a one-way relationship in which depressed mood always precedes ED symptoms. For instance, longitudinal research with over 1000 adolescent girls revealed that body dissatisfaction, dietary restraint, and bulimic symptoms predicted later onset of depression even after controlling for baseline negative mood in initially non-depressed participants [24]. Other studies also have yielded similar results suggesting that eating- and weight-related disturbances increase risk for subsequent depression [25,26]. Adding further complexity, a study conducted by Abrantes and colleagues [27] found that dieters at an adolescent inpatient psychiatric program were more likely to meet criteria for an ED, reported greater levels of distress, and had higher rates of major depression in the previous year compared to non-dieters.

Research also has implicated a role for low self-esteem in both EDs and depression. For instance, in a large-scale prospective study examining the relation between eating and weight-related disturbances, Rawana [25] found that self-esteem moderated the prospective relationship between trying to lose weight and depression. In addition, low self-esteem has been identified as a risk factor for bulimia nervosa [18], and high self-esteem was found to be protective in the development of later EDs in a large-scale study of Spanish adolescents [28]. Isomaa and colleagues [23] also found that their high-risk dieting group (i.e., feeling fat dieters and depressed dieters) reported lower self-esteem and more depressed mood than lower risk dieters.

Another potential target for public health initiatives is bullying, including weight-related teasing. Research supports the influence of bullying both in the development of EDs and depression. For instance, using a retrospective design, Striegel-Moore, Dohm, Pike, Wilfley, and Fairburn [29] found that bullying was significantly more common among both black and white women with binge ED compared to healthy controls. Importantly, the rate of bullying was not elevated relative to a psychiatric control group, of which 50% were diagnosed with a mood disorder. This provides evidence that bullying is a nonspecific risk factor for the development of eating pathology and that it also increases risk for other forms of mental illness.

With regards to prospective research on bullying and teasing, Haines, Neumark-Sztainer, Einsenberg, and Hannan [30] used a longitudinal design and found that weight related teasing at baseline significantly increased risk for binge eating with loss of control in boys as well as use of unhealthy weight control behaviors. Among girls, weight-related teasing significantly increased risk for becoming a frequent dieter. Although the literature on bullying and later onset of depression is somewhat mixed [31], recent longitudinal research indicates that among youth at risk for later suicide (e.g., due to baseline suicidal ideation or elevated depression) bullying was associated with increased suicidal ideation and functional impairment at 2-year follow-up [31].

As noted above, a comprehensive review of all shared variable risk factors is beyond the scope of this article. It is worth noting that a variety of risk factors may increase risk for many forms of psychopathology, including EDs and depression. For instance, both childhood sexual abuse and pregnancy complications (including pre-term birth/low birth weight) may increase risk for a number of psychiatric disorders including EDs [18,29] and depression in some individuals [32-38]. Such risk factors may be considered potential future targets for changing the course of EDs and depression. Because we are not aware of public health initiatives to influence these risk factors with the intention of reducing EDs/depression, we do not focus on them in this article.

### Public health approaches: programs and campaigns

Although we are not aware of any public health initiatives aimed at EDs and depression on the scale of some of the public health campaigns described above (e.g., immunizations), there are a number programs/campaigns in which one can recognize key the elements of standard public health approaches. Below, we review a series of approaches that are either particularly noteworthy because of their scale or empirical support. We also examine approaches that are simply very common. We do not claim that this is a comprehensive review of all programs in existence as there are many (likely hundreds) of regional initiatives across many countries that use approaches similar to what is described a below.

All of the programs below aim at some level to reduce risk factors for EDs, if not EDs per se. Moreover, because all of the programs below target nonspecific risk factors that are shared with depression, theoretically they should improve the course of depression *if* they sufficiently impact the targeted risk factor. It is important to note that changing risk factors for EDs and depression has proven more challenging than many expected and one problem that will be highlighted in this review is the lack of adequate evidence for the effectiveness of many approaches. Moreover, successful alteration of a risk factor may or may not alter the course of the end point disorder of interest (i.e., EDs, depression). Thus, ultimately risk factor reduction studies will need to be followed by rigorous research aimed at determining whether or not the courses of EDs and depression are in fact altered. As can be seen from the review below, this final step has yet to be taken in virtually all of the public health approaches.

Lastly, in some cases, researchers, clinicians or organizations may not be highly motivated to determine whether or not the course of endpoint disorders (i.e., EDs and depression) is in fact changed if the risk factor itself holds sufficient interest. For instance, below we describe a public health initiative by Dove and the World Association of Girl Guides and Girl Scouts (WAGGGS). The primary aim of this initiative is to increase body confidence and self-esteem. Changes in depression or EDs would, of course, be appreciated, but they are not a primary goal of this program. Such initiatives seek to simply reduce risk factors for the sake of reducing distress associated with the risk factors.

The Institute of Medicine (IOM) differentiates three types of prevention approaches: universal, selective, and indicated programs [39]. Universal programs target all individuals in a population regardless of risk status. Universal programs are distinct from selective prevention programs, which target individuals believed to be at high risk for developing a particular mental disorder based on the presence of identified risk factors. Indicated prevention programs target individuals with symptoms of a mental disorder but who do not meet the full criteria for a disorder. Note that this categorical organization of prevention programs is imperfect given that risk is a continuous variable and because many populations that are broadly considered high risk still consist of a mix of both higher- and lower-risk individuals. For instance, one can argue that any program that targets females could be categorized as selective given that female gender is a significant risk factor for both depression and EDs. Such labeling, however, may obfuscate significant differences between programs that aim to reach all females (i.e., program is universally delivered to both low and high body dissatisfied females) versus programs that are designed only to reach the higher risk females (e.g., only those with high body dissatisfaction).

As noted above, public health initiatives typically seek to focus on the health of an entire population versus focus on a small subset. For this reason, in this paper we only focus on initiatives that have been implemented on a universal/selective basis (e.g., to all women in a given population) versus selective/indicated basis (e.g., self-selected women with elevated body dissatisfaction or women showing early symptoms of EDs). To date no universal/selective study has been adequately powered or appropriately designed to assess true prevention effects, in large part because these studies were generally conducted with less grant support. We define true “prevention effects” as demonstrating that a given program reduces the onset of clinically significant EDs and depression (e.g., cases of DSM-IV bulimia nervosa and major depressive disorder) relative to a control condition. In the absence of true prevention effects, most studies are designed to reduce variable risk factors and continuous measures of ED and depressive symptomatology. Presently, despite decades of research, only three programs (*Student Bodies*, the *Body Project*, and a *Healthy Weight Intervention*) have yielded what could be referred to as true prevention effects with respect to EDs. To our knowledge, no studies have examined whether or not onset of clinical depression was also changed, although reductions in continuous measures of negative affect have been found. The two studies that demonstrated the above three EDs prevention programs could produce true prevention effects were both selective/indicated studies [40,41], and thus are not reviewed here. Instead, we review public health approaches and campaigns that have targeted shared risk factors for EDs and depression.

### Public health approaches targeting body dissatisfaction

Programs focusing on body dissatisfaction are appealing for a number of reasons from a public health perspective. First, body dissatisfaction is exceedingly common particularly among females [42], and as noted above it is a variable risk factor for an array of negative outcomes, including EDs and depression. Thus, at least theoretically, wide scale and clinically significant improvement in body dissatisfaction should improve the mental health (e.g., eating disorders and depression), and potentially the physical health, of a large percentage of the population. As noted above, however, this assumption has yet to be to be adequately tested. Below we review a number of different public health related approaches to improving body dissatisfaction.

#### The *body project*

Developed by Stice and colleagues, the *Body Project* is based on the dual pathway model of EDs described above, and utilizes a cognitive dissonance-based approach. A well-known psychological phenomenon [43], dissonance is the uncomfortable psychological state that occurs when an individual’s beliefs and actions are misaligned. To reduce psychological tension, individuals typically alter beliefs to align with behavior. In the *Body Project*, participants voluntarily speak and act against the thin-ideal, a behavior which is inconsistent with any previously held pro-thin-ideal beliefs. Theoretically, this leads participants to reduce thin-ideal internalization, which decreases body dissatisfaction and subsequently dietary restraint, depressed mood and ED symptomatology. This intervention has been branded under a variety of names (e.g., *Sorority Body Image Program*, *Reflections*: *Body Image Program*^®^; *Succeed Body Image Programme*); to avoid confusion and because it was originally published under the title of the *Body Project*[44,45], we use this label.

The *Body Project* is supported by significant high quality research when implemented in a selective/indicated fashion [40,46-49]. From a public health perspective, however, what distinguishes the *Body Project* from *Student Bodies* and the *Healthy Weight Intervention* has been the relatively extensive testing, dissemination and implementation of the *Body Project* on a more universal/selective basis using community providers. The use of low cost community providers is often critical for public health approaches given problems with scaling interventions to reach larger populations if interventions must be implemented by highly trained (i.e., expensive) providers. Thus, task shifting to less trained and less expensive providers fits a public health perspective [50]. Community providers also can spread the message of a program within the community even when a program is not formally being run.

In a series of studies, Becker and colleagues [51-53] investigated if the *Body Project* remained effective when it was delivered universally to members of a university sub-community (i.e., local sororities) by peers on a mandatory basis. These studies had the potential to identify key weaknesses in the *Body Project* from a public health perspective. For instance, if the *Body Project* only worked when implemented on a voluntary basis, by more highly educated providers, and with only higher risk participants this would have undermined the likely scalability of the intervention.

Results from three randomized controlled trials (RCTs [51-53]) indicated that the *Body Project* could be implemented on a mandatory basis by peer-leaders with significant changes in thin-ideal internalization, body dissatisfaction, dietary restraint, and ED pathology. Moreover, the *Body Project* yielded benefits for both lower- and higher-risk participants whereas an alternative intervention only benefitted higher-risk participants [52]. Lastly, results indicated that at 14-months, the magnitude of effects was similar for this delivery mode of the *Body Project* as compared to selective/indicated, voluntary delivery with more highly educated providers; the *Body Project* in this format also reduced negative affect, which included depressed mood [53]. No other public health approach has amassed this level of scientific support.

The Becker et al. studies were not only RCTs, but also relied on research methodology known as community-based participatory research (CBPR). CBPR is commonly used in public health approaches, with the goal of ensuring that interventions designed to address the health needs of communities are implemented in partnership with community leaders and/or members [54]. In CBPR, researchers engage community partners in an egalitarian manner that truly shares power and decision making with the aim of integrating knowledge about health problems [54]. CBPR also aims to improve problems solving, build on community strengths, and facilitate the creation of sustainable programs [54]. For instance, in the above studies, with the exception of Becker, the research team consisted of community members. In addition, sorority leaders and members played a key role in determining how the *Body Project* should be implemented.

CBPR methods also played a significant role in a 7-year *Body Project* dissemination effort throughout North America [55]. In 2005, having learned of the partnership between Becker and the local sororities at her university, the Delta Delta Delta fraternity (Tri Delta) began piloting the *Body Project* on a national scale. For readers outside North America, sororities are largely North American institutions created by early female pioneers in academia with the aim of providing community and a sense of belonging.

Between 2005 and 2012, Tri Delta and Becker disseminated the *Body Project* to over 100 campuses within the United States and Canada [55]. The primary vehicle for dissemination involved the creation of a “*Body Image Academy*,” a 2-day training workshop. University professionals were trained as peer-leaders trainers in a “train-the-trainer” model that allowed for greater scalability at their home institutions. Although *Body Image Academy* is no longer active, many of the universities that sent professionals continue to implement the *Body Project* on an annual basis. The *Body Image Academy* model also was adopted by the Succeed Foundation in its efforts to disseminate the *Body Project* throughout the United Kingdom via universities.

To date, the *Body Project* has been tested almost exclusively with secondary school and university-aged participants. Recent evidence, however, suggests that the dissonance approach may work with younger ages when appropriately adapted. For instance, Halliwell & Diedrichs [56] found that a dissonance-based approach reduced thin-ideal internalization, body dissatisfaction and increased resilience to negative media in 12–13 year old girls. This is an important study from a public health perspective as schools are often considered prime settings for implementing public health programming.

Most recently, Dove and WAGGGS used the *Body Project* as the foundation for a new initiative which aims to either directly or indirectly reach 3.5 million girls, ages 7–14, within the WAGGGS global network. In consultation with Becker, Stice and Diedrichs (who collaborated with the Succeed Foundation and their *Body Project* efforts) WAGGGS and Dove developed the dissonance-based program, “*Free Being Me*,” as part of Dove’s global campaign to improve body confidence in girls and women [57]. This represents a very important step for Dove in that their previous public health initiatives were less evidence-based (see below). The Dove/WAGGGS effort also builds off the Tri Delta/Succeed Foundation train-the-trainers model as part of their global dissemination effort [58]. To our knowledge, *Free Being Me* represents the largest organized effort to disseminate evidence-based body image programming worldwide and as such will be an important program to watch. Still, we must sound two cautionary notes with regards to this effort. First, as noted above, limited research has investigated the effects of a dissonance program in younger girls. Importantly, however, limited research currently exists supporting *any* program in this age group. As such, Dove/WAGGGS are acting in the spirit of an evidence-based approach in seeking to adapt the program with the greatest volume of support in reducing body dissatisfaction in females. In addition, the extensive scale of the project will undoubtedly raise new logistical challenges and it will be interesting to see how these are addressed by Dove/WAGGGS over time.

### Media literacy programs

#### Media smart

##### Australian program

Media literacy programs such as *Media Smart*, like several other programs described below, seek to influence a risk factor for body dissatisfaction with the aim of thereby reducing body dissatisfaction and creating a cascading effect that impacts EDs and depression. Media both influences and reinforces prevailing social norms, beliefs, and attitudes about appearance, and the effects of media exposure on body image have been well documented [59-61]. More specifically, findings from meta-analyses indicate that even brief exposure to media that portrays the thin-ideal of beauty [61] increases body dissatisfaction, which, as noted above, is a potent a risk factor for EDs and depression.

Several recent studies [62-65] employed a universal approach to evaluate if *Media Smart* was effective in reducing ED risk factors in a young-adolescent, mixed sex audience. For instance, *Media Smart* was evaluated in a controlled efficacy trial with assessment of ED risk factors at baseline, post-intervention, 6 month, and 2.5 year follow up time points (*N* = 540 girls and boys in the 8^th^ grade). The authors concluded that media literacy reduced shape and weight concern, body dissatisfaction, ineffectiveness, and depression in a universal, young adolescent population of girls and boys. It should be noted, however, that reductions in shape and weight concern among girls were only present at 2.5 year follow up when attrition had reached approximately 46%. Also, a preliminary controlled efficacy trial of *Media Smart* with adolescent girls 15 years of age demonstrated that *Media Smart* was comparable to a control group and less effective than a perfectionism-focused prevention intervention [65]. In this trial, *Media Smart* did not improve weight and shape concern or any other ED risk factors in this population. These results suggest that replication trials are needed to fully ascertain the effectiveness of *Media Smart* with adolescents.

Recently, depression was examined as a moderator of the impact of the *Media Smart* program [66]. Results indicated a reduction in ED risk factors (shape and weight concern, media internalization, dieting) for participants with high depression, and a reduced rate in development of ED risk factors (shape and weight concern) for participants with low depression. In sum, *Media Smart* is supported by research that suggests that it can be useful for a universal audience that is under the age of 15 who is at both low- and high-risk of developing an ED. That said, replication trials, particularly independent replication trials, in this line of research are needed with young individuals, as it has been suggested that ED risk factor constructs may be less stable in young adolescents, as opposed to older adolescents [67].

#### Media smart

##### United Kingdom (UK) program

*Media Smart UK*[68] is a UK based media literacy initiative that consists of one lesson aimed at improving body image. Some other components of this program also are known as *Be Adwise. Media Smart UK*, which was launched in 2002 and provides free educational materials, targets primary school children (children aged 7–11) and aims to aid children in understanding and interpreting advertising. This non-profit initiative was funded by the for-profit advertising industry, which collaborated with media literacy specialists. The body image lesson, which targets 10–11 year olds and was launched in 2011, was created to align with the UK government *Body Confidence Campaign* (see below). Since its inception, the *Media Smart UK* body image lesson has been downloaded over 1500 times [69].

To our knowledge, only one study has evaluated the effectiveness of the *Media Smart UK* body image lesson. Diedrichs and colleagues randomly assigned 204 boys and girls to either the body image lesson or a waitlist control [69]. Results indicated no benefit of the *Media Smart UK* body image lesson over the wait list control condition for all body image variables; in fact the control group outperformed *Media Smart UK* in weight esteem for boys. In summary, *Media Smart UK* not only lacks evidence for its benefits, but one study provides support for the interpretation that the program simply does not work at altering body image. Although well-intentioned, *Media Smart UK* provides a cautionary example of the problem with distributing public health type approaches prior to rigorous testing.

### Labeling/banning of photo retouching or very underweight models

Recently, significant attention has been focused on the effects of photo retouching in popular media (e.g. magazines and online media) to “correct” flaws in the appearance (e.g. imperfections in eyes, wrinkles, skin tone, hair) and/or body shape and size of individuals featured in the media [70]. Concerns generally focus on the notion that the result of “retouching” presents an extremely unattainable image for individuals to achieve, thus increasing body dissatisfaction. Thus, like media literacy programs, efforts targeting photo retouching aim to modify a risk factor of an ED/depression risk factor (i.e., body dissatisfaction) with the aim of altering body dissatisfaction and associated negative outcomes.

Several countries, including France, Australia, and the United Kingdom [71-73] have proposed or are currently initiating efforts to add disclaimers or warning labels to media images retouched using Photoshop. The most far reaching example of this occurred in Israel in 2012 when the Adatto Photoshop bill became law. This law bans the use of models with a Body Mass Index (BMI) of less than 18.5 and requires labeling of photoshopped images [74]. These actions fit with policy approaches in public health. Parallel initiatives from other areas include putting warning labels on items/substances that are harmful (e.g., cigarettes) or to simply ban said stimuli (e.g., USA Food and Drug Administration effort to potentially remove artificial trans fats in processed foods) in an effort to reduce consumption of those items [75,76].

Research on the likely benefit of these efforts has yielded mixed outcomes [77,78]. In a study conducted by Slater and colleagues [77] participants who viewed images with a warning label reported lower levels of body dissatisfaction than participants who viewed the images without a warning label, irrespective of the degree of the internalization of the thin-ideal. In contrast, Alta, Thompson, & Small [78] found no benefit on body dissatisfaction or intent to diet with either a disclaimer label (“Retouched photograph aimed at changing a person’s physical appearance.”) or a warning label (“Warning: Trying to look as thin as this model may be dangerous to your health.”) relative to control conditions. In sum, research examining the effects of labeling media images on female’s body dissatisfaction and mood is mixed. Further, at this time it is unclear if such labeling will significantly impact body dissatisfaction on a population level, let alone EDs and depression. However, we should note that to date studies have been small, investigating the effects of short term exposure to such stimuli. To our knowledge no research has investigated longer term effects of a markedly changed media environment such as might result from the Israeli government’s efforts; indeed we are not sure how such research could be conducted in a controlled, experimental design. Thus, the mental health benefits of environmental changes resulting from government-based policy with respect to media images likely will have to be studied longitudinally as country-wide social experiments.

### Campaigns promoting positive messaging aimed at body image and/or sexism

Body image and gender activists have designed and implemented numerous “campaigns” aimed at promoting positive body image and/or addressing sexism. Typically, not unlike other public health awareness campaigns, these efforts are largely not classified as “interventions” or “programs” but simply as campaigns to increase awareness around certain topics (e.g. body acceptance, body appreciation, building body confidence, reducing sexism in the media). In some cases, activists may develop interventions or workshops aimed at promoting their causes in schools as sub-elements of their campaign. In many cases, such campaigns and associated curricula are highly adopted by the general public (e.g., *The Representation Project*), despite lacking empirical support for their effects on the targeted issue. We outline several of these efforts below.

We note that campaigns vary in the degree to which they are explicit about the intended effects of their efforts on body image as well as the degree to which they state or imply that their campaign will ultimately affect variables such as EDs and depression. For instance, although neither *The Representation Project* nor the *Campaign for Body Confidence* explicitly state they will alter body image or the course of eating disorders, both do explicitly describe negative mood and eating disorders as potential results of sexism/objectification and pressure to conform to unrealistic images (see *Miss Representation* movie; and http://campaignforbodyconfidence.wordpress.com/about/). Thus, there is an implication that correcting said forces will reduce onset of EDs and depression. Other campaigns (e.g., *Love your Body Campaign* by the National Organization for Women) describe predicted effects of improved body image as an outcome (e.g., “feeling less stressed about appearance” at http://loveyourbody.nowfoundation.org/whatsitallabout.html). Lastly, as noted above with regards to regulating models and photoshopped images, some elements of campaigns may be very difficult to test whereas others (e.g., associated curricula) would be easier.

#### ***FAT TALK FREE WEEK***

In 2008, Tri Delta launched a worldwide advocacy campaign called *Fat Talk Free Week* (FTFW). Coined by Nichter and Vuckovic [79,80] fat talk is a term that refers to gendered dialogue between females that reinforces the thin-ideal standard of female beauty. Research indicates that as little as 3–5 minutes of fat talk can significantly increase body dissatisfaction [81].

FTFW was designed as a social marketing campaign aimed at increasing awareness of fat talk and its harmful effects. FTFW also promoted Tri Delta’s *Body Project* dissemination effort. Since its launch, FTFW has been implemented in dance studios, fitness studios, primary and secondary schools, retail stores, and universities throughout the United States and internationally.

To date, only one study has examined the effectiveness of FTFW. Garnett, Buelow, Franko, Becker, Rodgers, and Austin [82] investigated the effects of FTFW on a sample of university women. Some participants in the study attended FTFW events, whereas others did not. Results indicated that both samples showed significant reductions in self-fat talk, in body comparison, and on a measure of body dissatisfaction post-FTFW, possibly suggesting diffusion effects. Campaign saliency emerged as the strongest predictor of decreased fat talk, suggesting that the message worked best for individuals who found the message resonated with them. In summary, one very preliminary study provides some support for FTFW in reducing fat talk and impacting body dissatisfaction, but more research is needed before concluding the campaign achieves its aims. Further, there is no evidence that FTFW impacts the course of EDs or depression. To be fair, however, the campaign was originally designed as a marketing platform for the *Body Project* and was not expected to be sufficiently potent to impact EDs or depression.

#### ***THE REPRESENTATION PROJECT***

*The Representation Project* (formerly *Miss Representation*) is classified by its developers as “a movement that uses media and film content to expose injustices created by gender stereotypes and to shift peoples’ consciousness towards change” [83]. This effort began in 2011 with the release and promotion of the film, *Miss Representation*, a film aimed at highlighting sexist depictions of women in the media and the consequent effects on women’s ability to reach their potential (e.g. reduced upward mobility in the workplace; body dissatisfaction; lack of desire for leadership as girls age). This effort also includes a curriculum that is designed to boost media literacy in children in K-12^th^ grade. Although the curriculum does not explicitly target body image in its learning objectives, body dissatisfaction is a theme that emerges throughout the original film, in *The Representation Project*’s partnerships (e.g., *Endangered Bodies*), and on their website (e.g., the statistics page which includes information about EDs and body dissatisfaction) [83]. As such, *The Representation Project* is commonly accepted as attempting to impact body image as well as broader goals.

*The Representation Project* has received significant exposure at schools, universities, and public forums across the country and has successfully improved discourse about the damaging effects of media. To our knowledge, however, efficacy studies have not examined the campaign and, perhaps more importantly, the media literacy curriculum. As highlighted in the case of the *Media Smart UK* program, even well-meaning media literacy programs like *The Representation Project* curriculum may fail to have the intended effect and thus waste valuable school time. As such, research is urgently needed to investigate whether or not the effects of the curriculum, which is being used in 17 countries [83], justify its widespread dissemination.

#### ***CAMPAIGN FOR BODY CONFIDENCE***

The *Campaign for Body Confidence* is a positive body image advocacy initiative in the UK, led by a partnership between the UK government (i.e., the All Party Parliamentary Group on Body Image) and the Central YMCA [84,85]. The goal of this campaign is to promote greater body confidence in men and women, and to circulate psycho educational materials (digital and otherwise) about body dissatisfaction, dysregulated eating behaviors, unhealthy appearance-related behaviors (e.g., cosmetic surgery), and media literacy. Still in its beginning stages, the campaign provides facts and base rates about eating behaviors, societal appearance ideals, and the principles of body diversity and acceptance. Further, the campaign calls for body activists to organize and propagate further campaigns and events to tackle societal pressures for appearance ideals and for efforts to change policies regarding media messages. To our knowledge, no research has been published regarding the efficacy or effectiveness of participating in this campaign. One positive aspect of this campaign, however, is the collaboration and support of the UK government, which is unique to body image campaigns. Government involvement has been crucial in many other successful public health endeavors in other areas. Additionally, campaigns like the *Campaign for Body Confidence* and *The Representation Project* promote further activism and discussion related to body diversity and body acceptance. Lastly, the UK government has the capacity to push evidence-based programming for body image, though to date they have not aggressively done so as far as we can discern. In summary, although well-intentioned, without research investigating the effects of spreading positive messages via campaigns, the degree to which the *Campaign for Body Confidence* has the potential to impact the course of body dissatisfaction and ultimately EDs, or depression it is ambiguous at best.

#### ***NATIONAL ORGANIZATION OF WOMEN’S LOVE YOUR BODY CAMPAIGN***

The United States National Organization for Women (NOW) focuses on an overarching goal of achieving equality for women through education and litigation. NOW concentrates on a wide range of women’s rights issues including women’s body image. Through NOW’s *Love Your Body Campaign*, the organization challenges the message that society evaluates a woman’s value according to her willingness and ability to manifest current beauty standards [86]. The *Love Your Body Campaign* utilizes mainstream media (e.g. websites, Facebook, and twitter) to provide information about EDs and body image and to promote body image activism. NOW’s website offers a multiplicity of body image activism ideas that women can undertake in addition to projects that the organization develops such as “Let’s Talk About It” [86]. Although body image activism is one component of the *Body Project* program described above, NOW’s *Love Your Body Campaign* lacks empirical research supporting the efficacy of the campaign’s ability to improve women’s body image. In contrast to *The Representation Project* and *Media Smart UK*, however, we are not aware of any efforts by NOW to secure significant school time. Thus the potential cost of this program, even if ineffective, is substantially lower.

### Public health approaches targeting self-esteem

#### ***Dove workshops***

The Dove Self-Esteem Project (DSEP) [87], previously known as the Dove Self-Esteem Fund, is a program targeting girls aimed at improving body image and self-esteem, both of which targets are variable risk factors for EDs and depression. As part of the DSEP, Dove created the Dove Self Esteem ToolKit for girls [88]. The workshops are open to adolescent girls (ages 8–17 years) and their adult female mentors. The educational and interactive sessions focus on society’s creation of ideal images of beauty and promote the initiation of positive dialogue about beauty and self-esteem. Several incarnations of these workshops exist, including *BodyThink* in Australia which targets media literacy, self-esteem, internalization of the media ideal, body comparison and appearance teasing in young men and women. This self-esteem initiative by Dove also received input from BEAT (the UK EDs association), the Butterfly Foundation (Australia), and the Girl Scouts of the USA. This program has been disseminated throughout the UK in schools using the title of *BodyTalk*. In Australia, the Butterfly Foundation provided training to over 900 professionals for the dissemination of this program. As of June 2008, 40,000 adolescents (ages 10–17 years) had participated in the program [89].

To our knowledge, only one study investigated the efficacy of this widely disseminated program. Richardson, Paxton, and Thomson [89] conducted a study of 277 grade 7 students from 4 secondary schools in Australia. The adolescents in the intervention group participated in *BodyThink* during four 50-minute class periods while the adolescents in the control condition attended class as usual. In both adolescent males and females Richardson et al. [90] found no significant differences between the groups on measures of body dissatisfaction or dietary restraint and bulimic symptoms. The results largely demonstrate *BodyThink*’s lack of impact on body image or ED symptoms (depression was not assessed), and once again raise concerns about organizations widely disseminating media literacy programs prior to investing in the rigorous testing needed to determine if they work. As noted above, however, Dove has recently adopted a more evidence-based philosophy in its partnership with WAGGGS which is very encouraging.

#### ***Full of ourselves***

Yet another public health program targeting self-esteem, confidence, and self-acceptance is the wellness program “Full of Ourselves: Advancing Girl Power, Health, and Leadership” [90,91]. This program was designed as an EDs and obesity prevention program with a strong feminist approach that focuses on esteem-building, building leadership skills, and providing opportunities for both leadership and receiving mentorship. Two expected outcomes are improvements in the depression/ED variable risk factors, body image and self-esteem.

The program includes two phases; during the first phase, small groups of 11–14 year-old girls receive the program, 8 units total, delivered by adult women. Phase one employs an interactive structure with discussions, role-plays, and out-of-session activities (e.g. daily self-proclamations, letter-writing to advertiser or magazine about problems with material related to body esteem). In phase two, participants from phase one become peer leaders for younger girls, ages 8–11 years-old.

In a strong effort to assess effectiveness of the program, Steiner-Adair and colleagues [90] evaluated an implementation of *Full of Ourselves* in a partially randomized controlled trial across 24 schools in the Northeast United States comparing the program to an assessment-only control group. Results indicated that participants in the *Full of Ourselves* program reported increased knowledge (regarding health, weightism/appearance, and media literacy), as well as improved weight-related body esteem as compared to control participants through 6 month follow-up. Results did not suggest a difference between participants and controls in self-reported dysregulated eating behaviors (e.g. skipping meals). Of note, although results indicated an increase in knowledge and body esteem, there were no significant program effects on measures of body dissatisfaction, a critical and established risk factor for EDs and depression. A notable strength of the *Full of Ourselves* program is the ability to implement in school systems on a large scale while maintaining modest effectiveness at reducing some risk factors. Additionally, research assessing outcomes of participating in the program exists, which is an aspect that we have argued in this paper as necessary prior to large-scale implementation efforts. Although results of this program suggested modest improvement in some target variables further research is needed to investigate whether or not this program has a true prophylactic effect for onset of EDs, obesity, or depression.

### Public health approaches targeting bullying

#### ***Very important kids***

*Very Important Kids* (V.I.K.) is a school-based program designed to reduce weight-related teasing and unhealthy weight control behaviors (e.g., skipping a meal, taking diet pills) in children in grades 4 through 6. This multi-component program is aimed at producing change at both the individual level and the greater school/community environment [92]. A study evaluating the effectiveness and feasibility of VIK [92] found that this program was effective in reducing weight-related teasing in those schools that received the intervention compared to those serving as assessment only controls. Specifically, after controlling for baseline measures of overall teasing, this study found students in the intervention condition reported significantly lower odds of being teased about their appearance. Overall, intervention schools reported a 10% decrease in teasing from pre to posttest. Additionally, students who received the intervention reported significantly greater self-efficacy to change or impact weight-related teasing (e.g. stand up for a peer who was being teased) and a decrease in negative weight-related norms (e.g. talking about a peer’s weight). Somewhat less encouraging, V.I.K. largely failed to impact secondary outcomes such as body image concerns and unhealthy weight control behaviors. A major strength of V.I.K was the developers’ use of a CBPR approach for implementation; students in V.I.K. actively contributed throughout the course of the program, ensuring ideas and concepts were culturally relevant. As with other programs that target risk factors for the development of EDs and depression, it remains unclear if V.I.K will impact the course of these endpoint disorders. However, in contrast to some programs reviewed here, V.I.K. has not been widely disseminated ahead of testing.

### Public health approaches targeting eating disorder and obesity

Due to the overlap in risk factors for EDs and obesity (e.g., body dissatisfaction, depression, dieting, media consumption) researchers have called for programs that aim to prevent both EDs and obesity (e.g. Neumark-Sztainer et al. [93]). Given that obesity is considered a prominent public health target, we would be remiss in not touching on a few of these programs even though a more detailed review is beyond the scope of this paper. Two examples of this approach include *Planet Health* and *Life Smart*.

#### ***Planet health***

*Planet Health* is a two-year obesity prevention program for girls and boys in grades 6–8 that was tested in a large-scale school-based cluster RCT. Ten schools were randomly assigned to receive *Planet Health* or a control condition. The primary aim of *Planet Health* was to create change in four primary areas: reduce television watching to less than 2 hours per day, increase moderate and vigorous physical activity, decrease consumption of high-fat foods, and increase consumption of fruits and vegetables. Although the primary aim of *Planet Health* was to impact obesity, later analyses investigated effects of Planet Health on ED related behavior. Results indicated that in addition to reducing rates of obesity [94], *Planet Health* also reduced onset of purging behaviors amongst girls in the intervention condition [95]. To our knowledge no data indicates whether or not *Planet Health* impacted depressive symptoms. In summary, there is some limited evidence to suggest *Planet Health*, a public health program aimed at obesity, impacted one dimension of ED symptomatology, but it is unclear to what degree full syndrome EDs were impacted.

#### ***Life smart***

*Life Smart* consists of an eight session school-based program aimed at reducing the risk of both EDs and obesity in young adolescent girls and boys [96]. In a recent pilot study conducted by Wilksch and Wade [96], grade 7 girls and boys (*N* = 115) were randomly assigned to *Life Smart* or a control group. Risk factors were measured at baseline and post-intervention, which occurred five weeks after the delivery of the intervention. Results indicated that participants enjoyed the program and perceived it as valuable. Further, *Life Smart* showed promising results in reducing such ED/depression risk factors as shape and weight concern, body dissatisfaction, peer-teasing, and media internalization in girls. Boys who participated in *Life Smart* experienced no significant benefits [96]. Further research will be needed to determine if this program is capable of yielding true prevention effects.

## Conclusions

Public health approaches to disease prevention and health promotion have achieved many impressive outcomes in the area of physical health. Given the limitations of the expensive, expert treatment provider approach to reducing mental health suffering on a global scale [1,4], it is past time to investigate how public health approaches can further inform mental health programming. In this paper we focus on altering the course of EDs and depression.

The results of this review are both encouraging and discouraging in our opinion. On the encouraging side, research [51-53] increasingly supports the public health utility of one program (i.e., the *Body Project*) which has been found to actually reduce onset of EDs in selective/indicated research [40], and we are seeing increased efforts at dissemination of this program on increasingly larger scales. Further, more preliminary research supports several other programs in reducing at least one ED and depressive risk factor when delivered in a manner consistent with public health approaches (e.g., *Media Smart* (*Australia*), *V.I.K*., *Planet Health*; *Life Smart*). Additionally, there is some evidence that at least some of these programs also reduce depressive symptoms/negative affect. We also are encouraged to see that some major players in this area (e.g., Dove) are increasingly disseminating evidence-based approaches (including with schools) instead of widely disseminating untested programs. Lastly, we think that it is encouraging that EDs and associated risk factors (e.g., body dissatisfaction) finally appear to be on the radars of policy makers in at least some national governments.

On the discouraging or concerning side, most public health efforts to impact the course of EDs and depression, or the course of risk factors for these disorders, lack empirical support at best (e.g., *The Representation Project*; *Media Smart* (*UK*)), and those discussed in this paper are just the large scale examples of the types of programs that regional organizations and advocates frequently deliver in our experience. Thus, the overall landscape of public health approaches consists of a vast amount of time, energy and in some cases money being spent on programs that either have no demonstrated efficacy or, in the worst instances, actually have been shown to not work. One might ask whether or not this is in fact a problem if well-meaning individuals and organizations are at least trying to have an impact. We argue it is a problem and turn to evidence from another public health intervention from another area, namely substance prevention in the United States, to support our case. The Drug Abuse Resistance Education (DARE) program is a widely distributed substance abuse prevention program that launched in the early 1980’s without prior testing. In 1998 evidence emerged that the DARE program was ineffective at best [97]. Although DARE has since received funding to update its programming, one must ask what was the cost of those years when an ineffective program was being widely distributed. The General Accounting Office of the US Government placed the price tag at approximately 2 million dollars in the year 2000 alone [98]. Yet, an independent economist [99] estimated that the real cost of DARE was approximately one billion dollars per year when all costs (e.g., lost classroom space/time, law enforcement officer time etc.) were taken into account. We consider this history a warning for advocates and organizations seeking to impact risk factors for EDs and depression using public health approaches. There are real (yet often hidden) costs to ineffective programs, not the least of which is that when schools and other community settings invest in ineffective programs they are not using their resources to implement more effective programs or alternative educational initiatives that could work.

We consider attempts to label photographs and ban models of a certain size as potentially positive. Research clearly supports the negative impact of thin-ideal media images on body dissatisfaction, and body dissatisfaction is an ED/depression risk factor. Thus it is understandable that people want to see if reversing the trend in media images could be beneficial; indeed it may be that if governments can create sufficiently large scale change, there will be benefits. At the same time, this appears also to be yet one more instance when public health approaches are moving very far ahead of the empirical literature. It would seem to us that a more logical approach would be to invest further in appropriate research before changing policy.

Lastly, even with the most promising programs, we still need more research. For instance, it is unclear if public health type dissemination of the *Body Project* will yield true prevention effects, particularly when the program is delivered on a very large scale. We also need additional research investigating best practices for dissemination and implementation of efficacious programs. In addition, no program has been demonstrated to impact the onset of both clinically significant EDs and depression.

In summary, although public health approaches offer much promise in changing the course of EDs and depression, in our opinion we need significantly more research to determine a) which efficacious programs can be widely disseminated, b) which campaigns actually have an impact on behavior, risk factors and/or EDs and depression, and c) which programs and campaigns should be judged ineffective and eliminated to conserve and better deploy resources. In addition, we suggest that greater restraint is needed by organizations and advocates so that there is less dissemination of untested programs, which have the potential to waste significant resources. We appreciate the desire by many advocates, organizations and even governments to intervene so as to reduce the burden of EDs, depression and associated risk factors. Yet, it is critical in resource tight environments to be cognizant of costs and to not assume that a desire to do something positive will in fact have a positive outcome.

## Abbreviations

ED: Eating disorder; FTFW: Fat talk free week; NOW: National Organization of Women; DSEP: Dove self-esteem project.

## Competing interests

Carolyn Black Becker is co-director of the Body Project Collaborative, a social entrepreneurship company aimed at facilitating dissemination and implementation of the *Body Project* program that is described in this paper. She also sits on the scientific advisory board of Emerge (see below under Tiffany Stewart). As noted in this body of this paper, she consulted with Dove and WAGGGS on the development of the *Free Being Me* program. Lisa Smith Kilpela is a trainer for the Body Project Collaborative. Tiffany Stewart is co-founder and chief scientific officer for Emerge (http://www.emergebodyimage.com/), a body image technology development company, which is a listed partner for *The Representation Project*.

## Authors’ contributions

All authors contributed to conducting the literature review, writing and editing of this paper. No section of this paper was exclusively produced by one author. All authors read and approved the final version of this manuscript.
